# 重组人血管内皮抑素与多西紫杉醇不同顺序用药调控移植瘤组织MMP及抗瘤效应观察

**DOI:** 10.3779/j.issn.1009-3419.2010.06.003

**Published:** 2010-06-20

**Authors:** 静 袁, 凯 李

**Affiliations:** 300060 天津，天津医科大学附属肿瘤医院肺部肿瘤内科，天津市肿瘤防治重点实验室 Department of Toracic Oncology, Tianjin Medical University Cancer Institute and Hospital, Lung Cancer Center of Tianjin, Tianjin 300060, China

**Keywords:** 重组人血管内皮抑素, 多西紫杉醇, 用药顺序, 基质金属蛋白酶, Recombinant human endostatin, Docetaxel, Administration sequence, Matrix metalloproteinase

## Abstract

**背景与目的:**

通过药物干预性动物实验，观察重组人血管内皮抑素（rh-endostatin）与多西紫杉醇不同顺序联合用药后MMP-2及相关因子的变化及其对移植瘤生长与血管生成的影响，探索两药联合的作用机制和最佳抗肿瘤方案。

**方法:**

建立肺腺癌A549荷瘤裸鼠模型，分两个阶段进行实验。第一阶段：将荷瘤小鼠随机分为重组人血管内皮抑素组（重组人血管内皮抑素400 μg·d^-1^，d1-d14）和多西紫杉醇组（多西紫杉醇10 mg·kg^-1^·3d^-1^，d1-d14）；第二阶段：将荷瘤小鼠随机分为同时用药组（重组人血管内皮抑素400 μg·d^-1^、d1-d35，多西紫杉醇10 mg·kg^-1^·3d^-1^、d1-d19）、先重组人血管内皮抑素组（重组人血管内皮抑素400 μg·d^-1^、d1-d35，多西紫杉醇10 mg·kg^-1^·3d^-1^、d16-d34）和模型组。实验中动态测量移植瘤体积，结束后以免疫组化方法检测MMP-2、TIMP-2、EMMPRIN表达并计数微血管密度（MVD）。

**结果:**

两单药组比较，重组人血管内皮抑素组MMP-2和EMMPRIN表达下调较多西紫杉醇组（*P*=0.024, *P*=0.081）明显，两组间TIMP-2表达无明显差异（*P* > 0.05）。联合组用药结束时，同时用药组与先重组人血管内皮抑素组的移植瘤体积小于模型组（*P* < 0.001, *P*=0.003），且MMP-2表达均明显下调、微血管数减少（*P* < 0.05），但同时用药组对肿瘤生长的抑制较先重组人血管内皮抑素组明显；与模型组相比，同时用药组TIMP-2上调（*P*=0.001）、EMMPRIN下调（*P*=0.018），先重组人血管内皮抑素组未见相似结果。

**结论:**

同时用药方案可以从TIMP-2、EMMPRIN两个环节下调MMP-2的表达，从而更好地抑制肿瘤生长。

## Abstract (en)

**Background and objective:**

The aim of this study is to observe the changes of MMP-2 and its regulators, and to investigate the mechanism of the two administration sequences of recombinant human endostatin (rh-endostatin) and docetaxel.

**Methods:**

The experiment was performed as 2 stages. Firstly, nude mice with xenograft tumor were randomized into 2 groups as rh-endostatin-treated group with rh-endostatin 400 μg·d^-1^, d1-d14 and docetaxel-traeted group with docetaxel 10 mg·kg^-1^·3d^-1^, d1-d14. Secondly, nude mice with xenograft tumor were randomized into 3 groups as concurrent administration group (rh-endostatin 400 μg·d^-1^, d1-d35, docetaxel 10 mg·kg^-1^·3d^-1^, d1-d19), endo-first group (rh-endostatin 400 μg·d^-1^, d1-d35, docetaxel 10 mg·kg^-1^·3d^-1^, d16-d34) and model group (positive control, mice burdened tumor without treatment). The volume of tumor was measured during treatment. Detection of the expressions of MMP-2, TIMP-2, EMMPRIN and the count of microvessel density (MVD) by immunohistochemistry stain examination were carried out at the end of experiment.

**Results:**

Compared with the docetaxel-treated group, more obvious down-regulation of expression of MMP-2, EMMPRIN (*P*=0.024, *P*=0.081) were observed in rh-endostatin-treated group. No significant difference was found in TIMP-2 expression between the 2 groups. In combined treatment groups, at the endpoint tumor volumes of concurrent administration group and the endo-first group were remarkably smaller than that in model group (*P* < 0.001, *P*=0.003). According to the administration procedure, concurrent administration inhibited tumor growth ber than endo-first treatment did. Both of the combined groups down-regulated the expression of MMP-2 and decreased microvessel density (*P* < 0.05). Compared with model group, the expression of TIMP-2 was upregulated (*P*=0.001) as well as EMMPRIN down-regulated (*P*=0.018) in concurrent administration group. Oppositely, the same results were not observed in the endo-first group.

**Conclusion:**

The schedule of the concurrent administration group could inhibit the tumor growth better, and it down-regulated MMP-2 expression through TIMP-2 and EMMPRIN, and thus slow down the tumor growth superiorly to another schedule of treatment.

1971年Folkman教授提出了抗血管生成治疗理论^[1]^，即肿瘤生长依赖新生血管，阻止其生成可阻断肿瘤营养供应。近年来，一些抗血管生成药物和化疗联合取得了较好疗效，但两种药物的各自特点比较、联合时的最佳用药顺序及分子机制等仍为亟待阐明的问题。本研究以荷瘤鼠为动物模型，采用重组人血管内皮抑素(恩度，rh-endostatin endostar)和多西紫杉醇(docetaxel)进行干预，通过观察瘤组织基质金属蛋白酶(matrix metalloproteinases, MMP)及其调控指标变化以及瘤组织微血管密度(microvessel density, MVD)和瘤体生长，对该问题进行了探讨。

## 1 材料与方法

### 1.1 材料

#### 1.1.1 实验动物及癌细胞株

BALB/c-nu/nu小鼠购自北京维通利华公司，4周龄-5周龄，雌性，体重16 g -18 g，于无菌条件下饲养。人肺癌A549细胞株由天津医科大学肿瘤医院免疫实验室惠赠。

#### 1.1.2 药物和试剂

重组人血管内皮抑素由先声药业提供，批号：20081004。多西紫杉醇，由江苏恒瑞医药股份有限公司提供，批号：08082311。基质金属蛋白酶及其抑制剂、诱导剂染色所用一抗均购自Santa Cruz公司，CD34抗体购自Abcam公司。二抗及DAB等购自北京中杉金桥生物技术有限公司。

### 1.2 方法

#### 1.2.1 细胞培养方法

A549细胞传代培养于RPMI-1640培养液中，37 ℃、5%CO_2_条件下贴壁生长。

#### 1.2.2 小鼠移植瘤模型的建立

调整细胞悬液浓度为5×10^7^个/mL，于小鼠腹股沟皮下注射0.1 mL/只。待瘤体大于150 mm^3^时，剪成直径2 mm的小块，接种至小鼠左侧腹股沟皮下形成移植瘤。

#### 1.2.3 动物分组与给药

第一阶段：接种10天、成瘤体积达到要求后将实验小鼠随机分成2组，8只/组，配制好的多西紫杉醇以注射用水稀释后腹腔注射，重组人血管内皮抑素以生理盐水稀释后于移植瘤对侧皮下注射给药。重组人血管内皮抑素组为重组人血管内皮抑素400 μg·d^-1^，d1-d14。多西紫杉醇组为多西紫杉醇10 mg·kg^-1^·3d^-1^，d1-d14。小鼠均于第14天处死、收获瘤块。

第二阶段：接种10天、成瘤体积达到要求后将实验小鼠随机分为3组，8只/组。重组人血管内皮抑素与多西紫杉醇给药方法同前，生理盐水皮下注射、注射用水腹腔注射给药。同时用药组为重组人血管内皮抑素400 μg·d^-1^，d1-d35；多西紫杉醇10 mg·kg^-1^·3d^-1^，d1-d19。先重组人血管内皮抑素组为重组人血管内皮抑素400 μg·d^-1^，d1-d35；多西紫杉醇10 mg·kg^-1^·3d^-1^，d16-d34。模型组为生理盐水100 μL·d^-1^，d1-d35；注射用水200 μL·3d^-1^，d1-d35。所有小鼠均于第35天处死、收获瘤块。

将瘤块浸泡于1 0 %福尔马林进行固定，随后制成蜡块，切片4 μm厚，用于免疫组织化学染色(immunohistochemistry, IHC)。

#### 1.2.4 疗效评价方法

测量瘤块长径a及短径b、2/w，计算瘤体积：V=(a×b^2^)/2，抑瘤率=(1-治疗组体积/模型组体积)×100%。

#### 1.2.5 免疫组织化学染色方法

采用Envision两步法检测各指标的表达：常规脱蜡至水，高温、高压修复3 min，3%H_2_O_2_避光孵育15 min，加一抗(工作浓度为1:150)，4 ℃过夜。二抗37 ℃孵育30 min，DAB显色，复染核，中性树胶封片。

结果判定：每张切片于高倍镜(×400)下随机选取8个-10个不重复的视野采集图像，使用Image Pro Plus 6.0分析图片，得到每张图片的积分光密度值(integrated optical density, IOD)，取平均值作为该切片的IOD(平均光密度和面积的乘积，可以全面反映蛋白表达量)。采用泛内皮标记物CD34标记血管并计算MVD，于镜下(×400)选3个血管最丰富的视野计数，取其均数，厚壁血管及直径较大者(> 8个红细胞直径)不计数。

### 1.3 统计学处理

以SPSS 15.0统计学软件分析。正态分布的计量资料以Mean±SD表示，两组间比较采用独立样本*t*检验，多组间比较采用方差分析方法(*ANOVA*)中的*LSD*法；不符合正态分布的资料以中位数表示，两组间比较采用*Mann-Whitney U*法。符合正态分布的双变量间的相关分析采用*Pearson*法，不符合正态分布的采用*Spearman*法进行统计。以*P* < 0.05为差异具有统计学意义。

## 2 结果

### 2.1 第一阶段(单药)

#### 2.1.1 肿瘤生长及MVD比较

两组瘤体积与MVD间差异均未见统计学意义(*P*=0.087, *P*=0.435)，但多西紫杉醇组肿瘤体积小于重组人血管内皮抑素组，MVD高于重组人血管内皮抑素组(图 1，表 1)。

**1:**
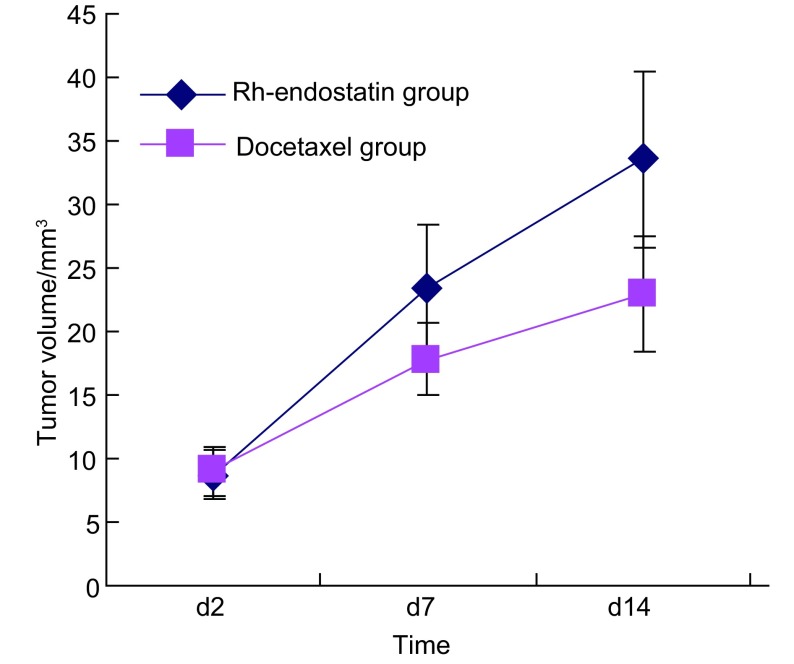
重组人血管内皮抑素组与多西紫杉醇组移植瘤生长曲线 Growth curves of transplanted tumor of Rh-endostatin group and docetaxel group

**1:** 重组人血管内皮抑素组与多西紫杉醇组的瘤体积、MVD比较 The comparison of tumor volume and MVD between the endostar group and the docetaxel group (Mean±SD, M)

Group	Tumor volume (mm^3^)		Difference (mm^3^)	*P*	MVD (count)	*P*
	Pre-therapy	Post-therapy				
Rh-endostatin group	7.67 (M)	33.58±13.79	24.88±14.22	0.087	11.97±3.95	0.435
Docetaxel group	8.98±3.84	22.94±9.11	13.96±9.59		14.71±8.06	

#### 2.1.2 各免疫组化指标比较

重组人血管内皮抑素组MMP-2、EMMPRIN表达低于多西紫杉醇组(*P*=0.024, *P*=0.081)，两组间TIMP-2表达未见明显差异(图 2，表 2)。

**2:**
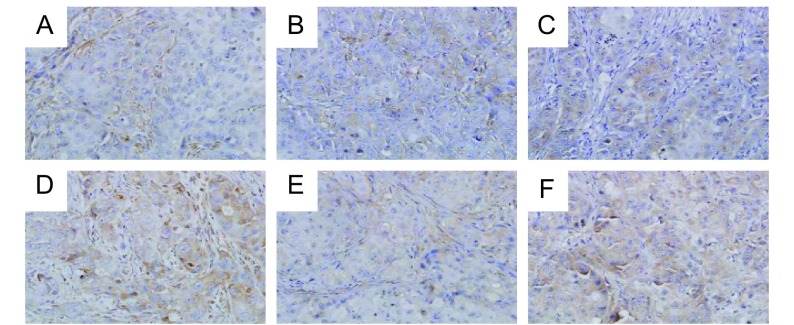
重组人血管内皮抑素组与多西紫杉醇组MMP-2、TIMP-2、EMMPRIN表达（IHC, ×400） The expressions of MMP-2, TIMP-2, EMMPRIN in the endostar group and docetaxel group (IHC, ×400). A, B, C: MMP-2, TIMP-2, EMMPRIN expression of the Rh-endostatin group in turns; D, E, F: MMP-2, TIMP-2, EMMPRIN expression of the docetaxel group in turns.

**2:** 两单药组MMP-2、TIMP-2、EMMPRIN表达情况比较 The comparison of the expression of MMP-2, TIMP-2, EMMPRIN between single-drug groups

Group	MMP-2			TIMP-2			EMMPRIN	
	IOD	*P*		IOD	*P*		IOD (M)	*P*
Rh-endostatin group	10 879.51±6 083.19	0.024		12 256.07±8 451.20	0.654		6 632.83	0.081
Docetaxel group	19 177.96±6 416.97			14 177.06±7 566.65			1 2111.8	

### 2.2 第二阶段(联合用药)

#### 2.2.1 肿瘤生长比较

治疗结束后两用药组肿瘤体积均小于模型组(*P* < 0.01)，两用药组间比较未见统计学差异(*P*=0.175)。治疗第18天至第29天内，同时用药组肿瘤体积均明显小于先重组人血管内皮抑素组(*P* < 0.05)，其早期抑制肿瘤生长作用更明显(图 3，表 3)。

**3:**
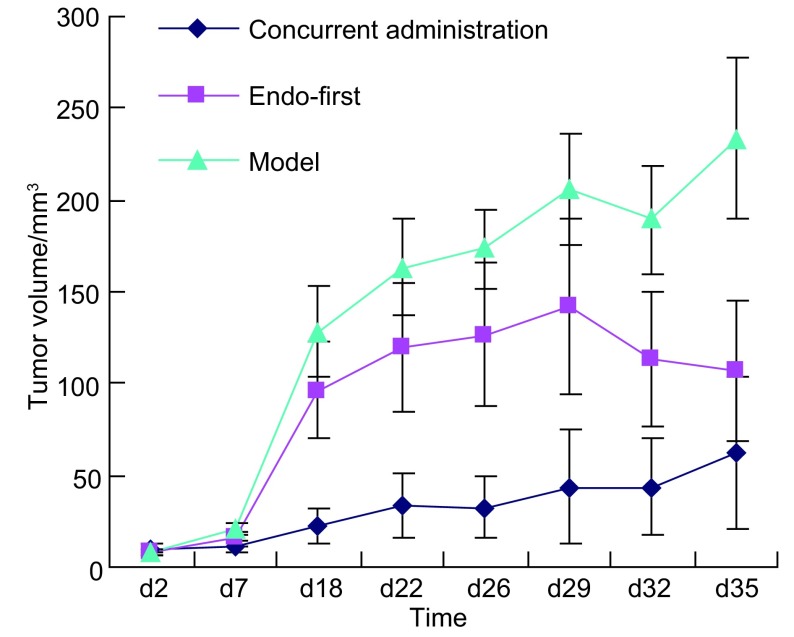
同时用药组、先重组人血管内皮抑素组、模型组的移植瘤生长曲线 Growth curves of transplanted lung tumor of the concurrent administration group, endo-first group and model group

**3:** 同时用药组、先重组人血管内皮抑素组与模型组治疗前、后瘤体积变化比较 Comparison of the changes of tumor volume during therapy among the concurrent administration group, endo-first group and model group (Mean±SD, *n*=8)

Group	Tumor volume (mm^3^)		Difference (mm^3^)	*P*	Inhibitory rate of tumor volume (%)
	Pre-therapy	Post-therapy			
Concurrent administration	7.14±2.58	66.55±41.25	59.40±41.52	0.175^a^	73.71
Endo-first	7.30±2.51	116.99±77.40	109.46±76.87	0.003^b^	51.55
Model	7.74±2.57	233.67±87.80	225.93±86.97	0.000^c^	0

#### 2.2.2 各组免疫组化指标比较

两用药组的MMP-2表达及MVD显著低于模型组(*P* < 0.05)，两用药组间比较未见统计学差异；同时用药组TIMP-2表达较先重组人血管内皮抑素组及模型组高(*P*=0.003, *P*=0.001)，EMMPRIN表达较模型组低(*P*=0.018)。先重组人血管内皮抑素组TIMP-2、EMMPRIN表达与模型组比较未见统计学差异(表 4，图 4)。

**4:** 同时用药组、先重组人血管内皮抑素组、模型组移植瘤的各组化指标染色结果比较 Comparison of the dyeing result among concurrent administration group, endo-first group, and model group (Mean±SD, *n*=8)

Group	MMP-2			TIMP-2			EMMPRIN			MVD	
	IOD	*P*		IOD	*P*		IOD	*P*		Count	*P*
Concurrent administration	34 126.16±22 074.36	0.987^a^		770 169.01±25 976.38	0.003^a^		6 541.44±4 491.06	0.297^a^		14.68±5.91	0.671^a^
Endo-first	34 023.76±19 855.44	0.002^b^		39 881.961±16 472.15	0.361^b^		10 866.82±6 008.53	0.100^b^		15.98±6.68	0.030^b^
Model	68 413.57±20 573.51	0.004^c^		32 231.73±7 984.01	0.001^c^		17 610.23±12 890.90	0.018^c^		22.79±4.78	0.014^c^
^a^: *P* value between the concurrent administration group and the endo-first group; ^b^: *P* value between the endo-first group and model group; ^c^: *P* value between the concurrent administration group and model group.											

**4:**
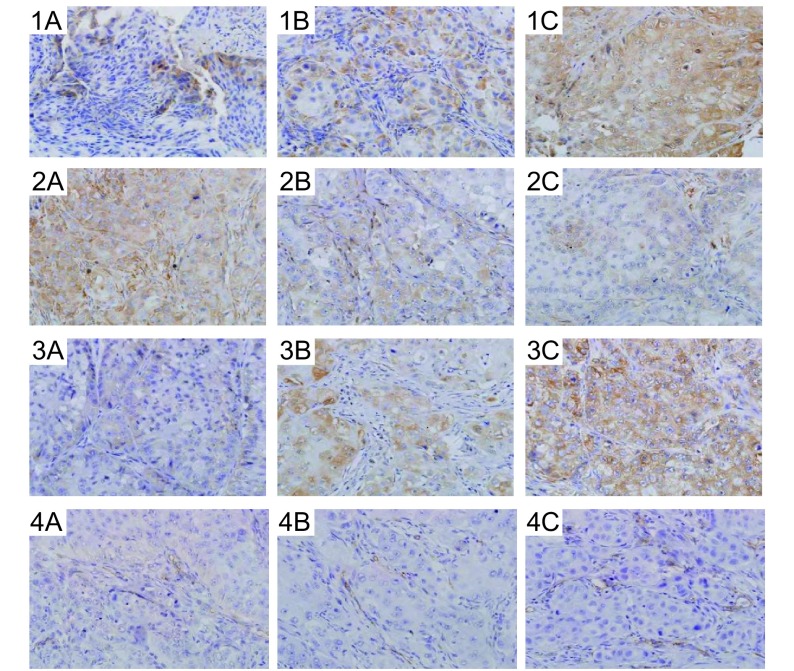
同时用药组、先重组人血管内皮抑素组和模型组各染色指标表达情况比较（IHC, ×400）。1-4依次为MMP-2、TIMP-2、EMMPRIN及MVD表达情况；A：同时用药组；B：先重组人血管内皮抑素组；C：模型组。 The comparison of the expression of each staining index among concurrent administration group, endo-first group, and model group (IHC, × 400). 1-4 were the expression of MMP-2, TIMP-2, EMMPRIN and MVD in turns; A: Concurrent administration group; B: Endo-first group; C: Model group.

### 2.3 相关分析

瘤体积与MVD呈明显正相关(*r*=0.529, *P*=0.017)，TIMP-2与肿瘤体积呈负相关(*r*=-0.558, *P*=0.015)。

## 3 讨论

研究^[2]^证实，抗血管生成与化疗药物联合治疗肺癌可以取得良好疗效，故常将以上两种药物联合使用。但如何找到二者的最佳搭配模式仍是争论热点。Huang等^[3]^认为，抗血管生成药物可将原本排列混乱的肿瘤血管网“梳理整齐”，畅通血流，令更多药物和氧进入，提高肿瘤对药物的敏感性。据此“血管正常化”假说，最佳次序应为抗血管生成先于化疗。但有些研究人员认为，“血管正常化”仅仅是一个短暂过程，不能对疗效产生重大影响；反之，先使用化疗大量杀伤肿瘤后再行抗血管生成治疗才可带来最大临床受益。目前以此模式完成的Avastin与化疗联合治疗肺癌的试验即获得了满意效果^[2]^。因此，亟需比较两种模式的不同疗效，并阐明机制，为确立最佳治疗方案提供依据。

MMPs是迄今为止发现的与肿瘤侵袭关系最为密切的一类蛋白水解酶，可降解细胞外基质(extracellular matrix, ECM)、增加毛细血管通透性，促进液体渗入组织间、压迫毛细血管、使其逐渐膨胀、迂曲，血管网排列也变得混乱。故在诸多血管生成因子中，其与“血管混乱化”的关系最为密切。理论上讲，抑制其表达或功能最可能将原本混乱的肿瘤血管重新排列“正常化”。

MMPs的表达受其抑制剂TIMPs(matrix metalloproteinase inhibitors, TIMPs)及诱导剂EMMPRIN的调节。MMP-2属于Ⅳ型胶原酶，在肺癌组织的表达明显高于正常肺组织^[4]^，其抑制因子TIMP-2是一种非糖蛋白，与MMP-2的亲和力很强，主要抑制其活性，但对MMPs家族其它成员也有作用。ECM的降解并不是由MMPs分泌的多少来决定，而是由MMPs/TIMPs的平衡决定^[5]^。MMP-2的诱导因子EMMPRIN(extracellular matrix metalloproteinase inducer, EMMPRIN)又称CD147，能诱导MMPs产生^[6]^、与肿瘤血管生成有密切关系^[7, 8]^。

Kim等^[9]^发现血管内皮抑素可与MMP-2前体蛋白(pro-MMP2)结合形成稳定复合体而阻止其活化、并抑制MMP-2和膜型MMP(MT1-MMP)的催化活性。也有研究^[10]^发现，重组人血管内皮抑素对MMP-2的抑制作用比对MMP-9的明显，可能是通过抑制ERK1/2磷酸化引起MMPs下调。多西紫杉醇也可明显抑制MMP-2^[11]^。

重组人血管内皮抑素由我国自主研发，其Ⅲ、Ⅳ期临床试验均证明它对化疗药物有明显的增效作用。我们选用其和多西紫杉醇进行药物干预，旨在比较二者的抗肿瘤特点，阐明联合用药时的最佳顺序及分子机制，为制定临床治疗方案提供依据。

实验第一阶段比较两药的各自效应，结果重组人血管内皮抑素组MMP-2、EMMPRIN表达低于多西紫杉醇组(*P*=0.024, *P*=0.081)，虽两组的瘤体积与MVD间差异未见统计学意义(*P*=0.087, *P*=0.435)，多西紫杉醇组肿瘤体积小于重组人血管内皮抑素组，MVD却高于重组人血管内皮抑素组；提示尽管前期研究已证实两药均可抑制MMP-2表达，但重组人血管内皮抑素通过下调EMMPRIN抑制MMP-2表达的作用更强，并可能更多地降低MVD。但这一“抑瘤潜力”却未能转化成良好效果、导致瘤体迅速生长。反之，尽管多西紫杉醇抑制MMP-2的作用可能弱于重组人血管内皮抑素，但由于其细胞毒作用而更有效地抑制了瘤体增大。因此，重组人血管内皮抑素抑制新生血管形成的能力虽然更强，但因对肿瘤细胞无显著杀伤、从而不能与化疗药物匹敌并取得良好疗效，利用其特点为化疗药物增效应更合理。因此，在第二阶段观察了联合用药效果及最佳治疗顺序。由于在第一阶段中重组人血管内皮抑素用药14天后观察到EMMPRIN、MMP-2明显下调，提示已达到稳定抑制MMP的状态，因此设定先重组人血管内皮抑素组重组人血管内皮抑素单药的作用时间为14天。

实验发现，两联合用药组MMP-2的表达均低于模型组，但同时用药组EMMPRIN表达明显低于先重组人血管内皮抑素组及模型组，并上调了TIMP-2表达，致使MMPs/TIMPs平衡改变、ECM降解减少，有效地降低了肿瘤血管通透性和组织间流体静压(interstitial fluid pressure, IFP)、减轻渗出液压迫微血管、达到“血管正常化”，使更多药物进入瘤体、为同时应用的化疗增效^[3]^，肿瘤体积也得以明显缩小。我们推测，同时用药组EMMPRIN和TIMP-2变化应归咎于早期联合用药迅速压制了肿瘤生长、使其分泌的肿瘤性促血管生成因子(tumoral angiogenesis factors, TAFs)减少，从而下调EMMPRIN的表达而上调TIMP-2表达(VEGF可抑制TIMP-2基因及蛋白表达^[12]^)。反之，先使用重组人血管内皮抑素尽管可能达到抑制MMP-2表达、促进“血管正常化”的作用，但因没有能有效杀伤肿瘤细胞的药物同期通过“正常化”的血管进入瘤体、发挥功效，致使肿瘤继续生长、产生大量TAFs(如VEGF)，全面促进肿瘤组织的血管内皮细胞增殖、活化及新生血管形成、增加血管通透性，这些作用可能部分、甚至全部抵消了MMP下调对肿瘤血管的作用，使其恢复为密集而混乱的状态。因此，虽然两种给药顺序均可通过MMPs环节起到抗肿瘤作用，但显然只有保证两药作用紧密衔接，方能充分发挥重组人血管内皮抑素的“增效潜力”。但也可能因为本组单药重组人血管内皮抑素给药时间较长(14天)，导致其作用未能与多西紫杉醇很好衔接而降低了效果。假如找到更合适的重组人血管内皮抑素作用时间窗，紧密衔接两药作用，或可达到更好疗效。

本研究从MMPs及其抑制剂、诱导剂的角度探讨了两种用药顺序的不同作用机制，为临床用药提供了一定的理论依据，也从基础研究角度验证了在ECOG4599、AVAIL、YH-16等国内、外临床试验中同时用药模式的合理性。但肿瘤生长是多步骤、多环节的复杂过程，对其它指标、通路的研究有待继续进行。
